# Anti-EGFR Reintroduction and Rechallenge in Metastatic Colorectal Cancer (mCRC): A Real-World Analysis

**DOI:** 10.3390/cancers14071641

**Published:** 2022-03-24

**Authors:** Martin Sebastian McCoy, Sebastian Wolf, Vera Struck, Niklas Thomas, Gabriele Husman, Stefan Zeuzem, Christine Koch, Jörg Trojan, Andreas Anton Schnitzbauer, Wolf Otto Bechstein, Oliver Waidmann

**Affiliations:** 1 Department of Internal Medicine I, University Hospital Frankfurt, Goethe University, 60590 Frankfurt, Germany; martin.schulz2@kgu.de (M.S.M.); vera.struck@gmx.de (V.S.); niklas-thomas@gmx.de (N.T.); stefan.zeuzem@kgu.de (S.Z.); christine.koch@kgu.de (C.K.); joerg.trojan@kgu.de (J.T.); 2 Department of Internal Medicine II, University Hospital Frankfurt, Goethe University, 0590 Frankfurt, Germany; sebastian.wolf@kgu.de; 3 Tumor Documentation, University Cancer Center, University Hospital Frankfurt, Goethe University, 60590 Frankfurt, Germany; gabriele.husmann@kgu.de; 4 Department of General and Visceral Surgery, University Hospital Frankfurt, Goethe University, 60590 Frankfurt, Germany; andreasanton.schnitzbauer@kgu.de (A.A.S.); wolf.bechstein@kgu.de (W.O.B.)

**Keywords:** chemorefractory metastatic colorectal cancer, anti-EGFR therapy, re-exposure, rechallenge, reintroduction

## Abstract

Background and Aims: In patients with *Rat sarcoma proto-oncogene* (*RAS*) wild-type metastatic colorectal cancer (mCRC), anti-epidermal growth factor receptor (EGFR) antibodies have been established in first- and further therapy lines. Due to limited treatment options upon disease progression, anti-EGFR re-exposure is increasingly employed in real-world oncology. The aim of this study was to assess clinical implementation and utility of anti-EGFR retreatment strategies in real-world mCRC patients. Methods: In this monocentric retrospective study, we included 524 patients with CRC and identified patients who received an anti-EGFR-based treatment as well as anti-EGFR rechallenge (progression on first-line anti-EGFR therapy) or reintroduction (discontinuation due to intolerance/toxicity/other). Results: In total, 143 patients received an anti-EGFR-based first- or second-line treatment, showing a similar overall survival (OS) compared to the non-anti-EGFR treatment group (38.3 vs. 39.6 months, *p* = 0.88). Thirty-three patients met the inclusion criteria for anti-EGFR re-exposure and were either assigned to rechallenge (*n* = 21) or reintroduction (*n* = 12) subgroups. The median FU after re-exposure was 45.8 months. Cetuximab and Panitumumab were used in 21 and 12 patients, respectively, and the main chemotherapy at re-exposure was FOLFIRI in 39.4%. Anti-EGFR re-exposure was associated with a distinct trend towards a better outcome (median OS 56.0 vs. 35.4 months, *p* = 0.06). In a subgroup comparison, reintroduction was associated with a higher OS and PFS in trend compared to the rechallenge (mOS 66 vs. 52.4, n.s., mPFS 7.33 vs. 3.68 months, n.s.). Conclusions: This retrospective study provides real-world evidence underscoring that anti-EGFR re-exposure strategies might benefit patients independently of the reason for prior discontinuation.

## Simple Summary

Colorectal carcinoma (CRC) is one of the most prevalent malignomas worldwide and a leading cause of cancer associated mobidity and mortality. While screening and therapeutic developments have improved long-term survival and cure rates, a considerable fraction of patients suffers disease progression and death. Monoclonal antibodies targeting the epidermal growth factor receptor (EGFR) are amongst the cornerstones of therapy in advanced-stage CRC. However, while prospective clinical trials have confirmed the benefit of including those agents into the regimes for advanced CRC, disease progression or therapy-related toxicities might require physicians to switch from anti-EGFR-based therapies to alternative treatments. With limiting options in later treatment lines however, re-exposure to anti-EGFR-based therapy regimes is a valuable option and evidence for this approach is limited. This real-world study from a large oncology center in germany includes data from more than 500 patients to underscore the benefit of anti-EGFR re-exposure in patients with advanced CRC.

## 1. Introduction

Colorectal cancer (CRC) is the fourth most frequently diagnosed cancer worldwide and a leading cause of cancer-related death [1]. In 2018, an estimated 881,000 patients died from CRC worldwide and more than 1.8 million patients were diagnosed with CRC [1]. Upon disease progression, approximately 50% of patients will develop metastasis, whereas 25% present with an advanced stage of CRC at initial diagnosis [2,3]. A majority of these patients is not suitable for initial curative resection. In recent years, corroborating evidence has established epidermal growth factor receptor (EGFR) inhibitors to provide a distinct clinical benefit for patients with *Rat sarcoma proto-oncogene* (*RAS*) wild-type metastatic colorectal cancer (mCRC) [4,5,6,7,8,9]. The EGFR pathway plays a critical role in mediating cancer cell proliferation and cell viability. Thus, monoclonal anti-EGFR antibodies cetuximab and panitumumab can be utilized to inhibit MAPK downstream signalling and are approved for treatment in combination with or after conventional chemotherapy [9,10,11,12]. However, in approximately 55–60% of cases, tumor biology is characterized by mutations in the EGFR pathway (*Kirsten rat sarcoma virus gene* (*KRAS*) exons 2/3/4, *Neuroblastoma RAS viral oncogene homolog* (*NRAS*), or *B rapidly accelerated fibrosarcoma gene* (*BRAF*) genes), which lead to constitute downstream activation and primary resistance to anti-EGFR therapy, rendering EGFR inhibition an inefficient therapeutic strategy [13,14,15]. Current treatment guidelines therefore recommend confirming RAS wild-type in all metastatic CRC (mCRC) patients prior to anti-EGFR therapy [3,16].

Anti-EGFR therapy has become a valuable treatment option for mCRC [5]. However, upon disease progression to the second-, third-, or fourth-line, the general outcome of patients is poor and available treatment options become more limited. Moreover, an increasing fraction of mCRC patients are ineligible for further cytotoxic chemotherapy, either because of poor performance status, severe adverse effects to prior chemotherapy (CT), or patients’ choice. Thus, depending on the response to initial cytotoxic and anti-EGFR-based therapy, anti-EGFR re-exposure is a promising approach in later line treatment, which is increasingly implemented into real-word oncology, although available data are limited. In regard to retreatment strategies, re-exposure after prior anti-EGFR discontinuation due to intolerance or toxicity (reintroduction) is distinguished from discontinuation due to mCRC progression under therapy (rechallenge) [17]. This concept of anti-EGFR rechallenge was evaluated as an approach to improve treatment efficacy in pretreated patients with mCRC progression [18,19]. However, robust evidence from larger prospective studies is missing to date and there are still areas of uncertainty, as real-world data on retreatment strategies are sparse and, moreover, several studies have insufficiently discriminated between reintroduction and rechallenge strategies. The aim of this retrospective study was to provide further evidence on implementation and clinical utility of anti-EGFR retreatment strategies in real-world mCRC patients.

## 2. Materials and Methods

### 2.1. Study Design and Population

In this single-center retrospective study, a real-life database was established including all patients diagnosed and treated with mCRC between 2011 and 2019. Patients were identified by using a computerized database ORBIS (Agfa HealthCare) and included in this analysis if they met the following criteria: histologic diagnosis of mCRC; clinical indication for first anti-EGFR therapy; and/or documented RAS and BRAF wild-type mutation status on acquired biopsy. Measurable disease according to the Response Evaluation Criteria in Solid Tumors (RECIST) version 1.1. *RAS*/*BRAF* status was determined by PCR and performed in tissue samples [20]. Rechallenge was defined as anti-EGFR-based therapy with treatment interruption upon disease progression and at least one intervening anti-EGFR-free therapy block before anti-EGFR re-exposure. In contrast, patients with reintroduction had no documented mCRC progression under first anti-EGFR therapy, but treatment was stopped due to other reasons, such as intolerance or toxicity.

### 2.2. Statistical Analysis

Normally, distributed data are expressed as the mean and standard deviation, and non-parametric data are expressed as the median and interquartile range. Distribution was assessed visually. Non-parametric testing for unpaired comparisons was performed via the Mann–Whitney U test for two groups and Kruskal–Wallis test for >2 groups. Survival rates were analyzed using the Kaplan–Meier method and compared through the log-rank test using the “survival” v3.2.13 and “survminer” v0.4.9 packages. Survival data are reported as median estimates with a 95% confidence interval (CI). Univariate and multivariate risk factor analyses were performed with the Cox regression model using the “survival” v3.2.13 package. The median follow-up was estimated using the reverse Kaplan–Meier estimator [21]. *p*-values < 0.05 were considered statistically significant. Statistical analysis was performed using R version 4.0.3.

## 3. Results

### 3.1. General Patient Characteristics

A total of 524 patients with a histological, radiological, and clinical diagnosis of CRC were identified, of which 435/524 (83%) presented an advanced union for international cancer control (UICC) stage IV disease with synchronous metastases. The most common site of metastasis at the initial diagnosis was hepatic, in 61.8% of patients, followed by other locations (including skeletal and central nervous system) in 19.2% of cases and pulmonary in 13.7% of patients.

**Figure 1:**
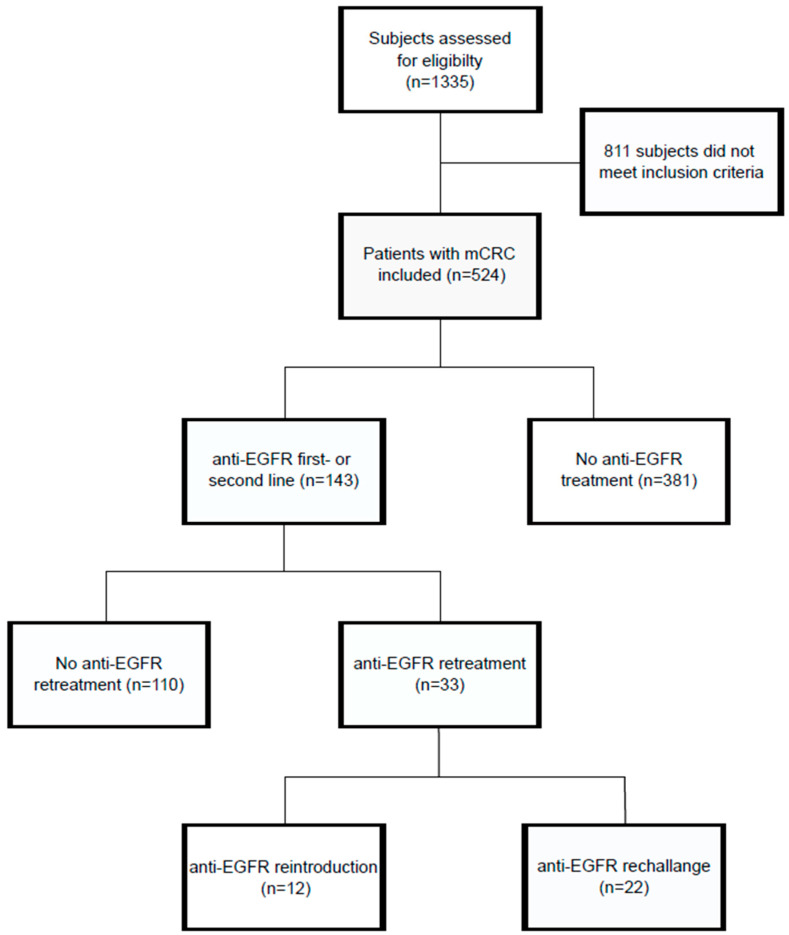
Flow chart depicting the study cohort and assignment into different study groups.

Of the patients, 143/524 (27.3%) received at least one anti-EGFR-based therapy line; and 33/143 patients (23%) received an anti-EGFR re-exposure after prior discontinuation. A CONSORT diagram displays the patient assignment into the respective study groups (Figure 1). Details on clinical characteristics are displayed in Table 1. The median age was 63 years, 306 patients (58.4%) were male, and 218 (41.6%) were female. The most frequent tumor localization was the left-sided colon, in 355 patients (67.7%).

In the overall cohort, CRC patients showed a median overall survival (OS) of 38.9 months (95% CI 34.2–44.6 months) and a median first-line progression-free survival (PFS) of 11.6 months (95% CI 10.2–13 months) (Supplementary Materials Figure S1). In 148/524 patients, a complete remission could be achieved by multimodal first-line therapy including chemotherapy, surgery, and local treatment, e.g., microwave ablation (mRFS = 13.7, 95% CI 11–16.9). Relapse-free survival (RFS) in patients with complete remission was 24 months (95% CI 22.1 to 29.9 months). In multivariate regression analysis, the most significant independent risk factors for poorer overall survival were synchronous metastatic disease (hazard ratio (HR) 1.89, 95% CI 1.28–2.80, *p* < 0.001) and age at diagnosis (HR 1.02, 95% CI 1.01–1.03, *p* < 0.001), while the primary tumor resection was independently associated with a better OS (HR 0.50, 95% CI 0.38–0.66, *p* < 0.001) (Supplementary Materials Table S1).

### 3.2. Anti-EGFR First-Line or Second-Line Treatment

In total, 143 patients with mCRC and a history of anti-EGFR therapy were identified. Clinical characteristics of the anti-EGFR-treated cohort are displayed in Table 1. Patients receiving anti-EGFR-based therapy were significantly younger (58.8 vs. 63.6 years, *p* < 0.001), and showed a lower rate of right-sided mCRC (20.3 vs. 36.6%, *p* < 0.001) and a slightly more advanced stage of disease at initial diagnosis (UICC IV 87.4% vs. 81.4%). Patients received a median of 4 cycles (IQR 4) of anti-EGFR-based therapy. In 64.3% of patients, anti-EGFR therapy was part of the first-line therapy, while in 35.7% of patients, anti-EGFR-based therapy was part of subsequent therapy lines. Notably, anti-EGFR-treated patients showed similar overall survival compared to patients without anti-EGFR-based therapy (median OS 38.3 vs. 39.6 months, *p* = 0.8). The median PFS in patients who received anti-EGFR-based first-line therapy was similar compared to patients with non-anti-EGFR-based first-line therapy (11.3 vs. 11.9 months, *p* = 0.2), as was the first-line median RFS (29.8 vs. 24.6 months, *p* = 0.1). Response rates to first anti-EGFR treatment are displayed in Supplementary Materials Table S2.

In a multivariate regression analysis, >1 anti-EGFR therapy block was significantly associated with a better overall survival (HR 0.51, 95% CI 0.30–0.85, *p* < 0.05) as was tumor resection at first diagnosis (HR 0.54, 95% CI 0.32–0.99, *p* < 0.05) and left-sided tumor localization (HR 0.64, 95% CI 0.37–1.09, *p* < 0.05) (Supplementary Materials Table S3). The presence of synchronous metastases at first diagnosis was associated with a significantly poorer OS in the anti-EGFR subgroup compared to patients with metachronous metastatic disease (HR 3.01, 95% CI 1.29–7.04, *p* < 0.05). In patients receiving anti-EGFR-based therapy, the median PFS of the individual last anti-EGFR-based therapy line was 7.89 months (95% CI 6.18–9.86).

### 3.3. Anti-EGFR Reexposure, Reintroduction and Rechallenge

**Figure 2:**
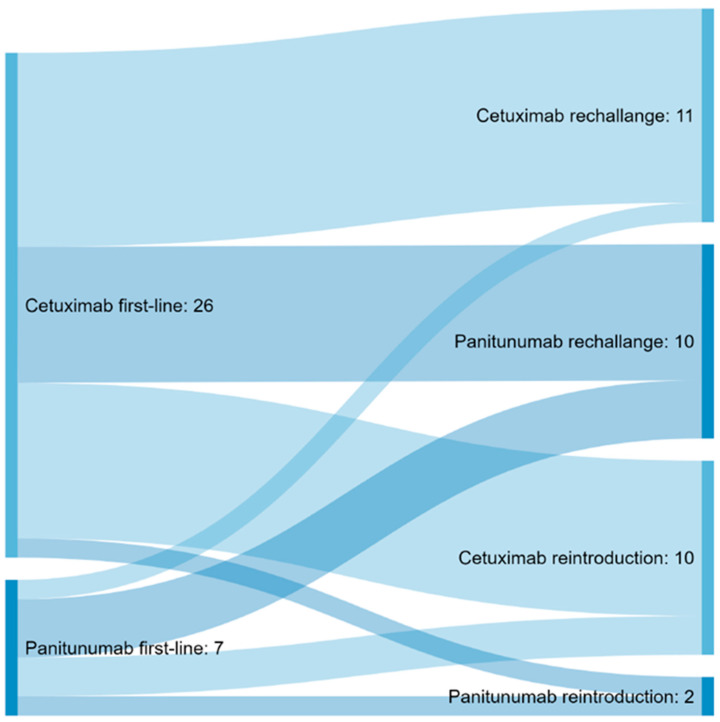
Sankey diagram displaying the proportional flow of patients between first anti-EGFR agent utilized in first- or second-line and anti-EGFR agent chosen for re-exposure.

Of the patients, 33/143 (23.1%) received anti-EGFR re-exposure after prior discontinuation (Table 2). At re-exposure, the main chemotherapy was FOLFIRI in combination with Cetuximab in 13/33 patients (39.4%), followed by FOLFIRI with Panitumumab in 9/33 patients (27.3%) (Figure 2, Supplementary Materials Table S4). The median anti-EGFR-free interval was 14.2 months, and the median follow-up after anti-EGFR re-exposure was 45.8 months.

**Figure 3:**
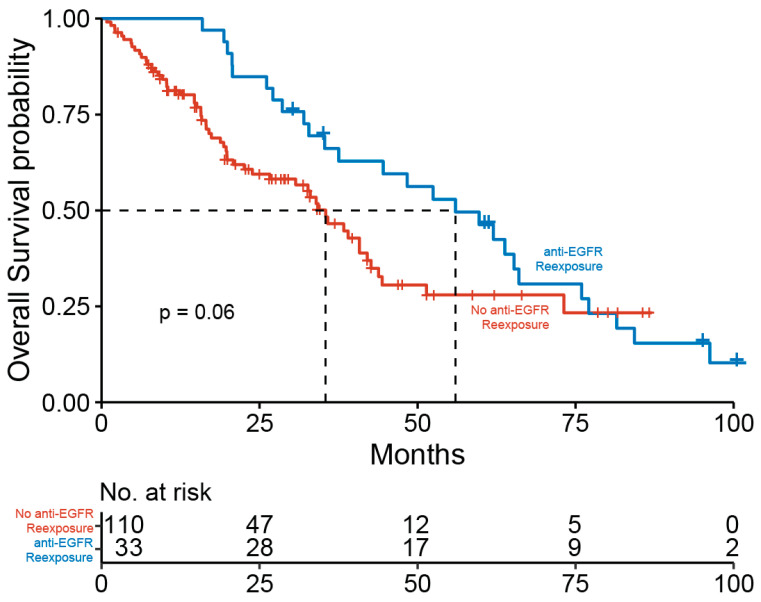
Kaplan-Meier estimator for the overall survival in patients receiving anti-EGFR-based therapy stratified by anti-EGFR re-exposure (blue) versus no anti-EGFR re-exposure (red).

Patients with anti-EGFR re-exposure showed a distinct trend towards a better overall survival compared to patients without re-exposure (median OS 35.4 vs. 56 months, *p* = 0.06) (Figure 3). Moreover, data showed good response rates in anti-EGFR retreated patients (complete remission (CR): 21.2%, partial remission (PR): 63.6%, stable disease (SD): 9.1%, progressive disease (PD): 6.1%). Response rates of patients after first anti-EGFR exposure and anti-EGFR retreatment are displayed in Supplementary Materials Table S2.

**Figure 4:**
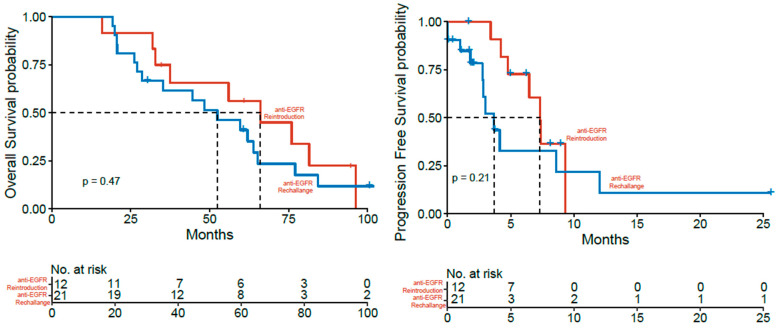
Kaplan-Meier estimator for overall (**left**) and progression-free (**right**) survival in patients with anti-EGFR reintroduction (red) and rechallenge (blue).

For further analysis, patients re-exposed to anti-EGFR were either assigned to a rechallenge (*n* = 21, progression under anti-EGFR therapy) or reintroduction (*n* = 12, anti-EGFR interruption due to other reasons) group. The analysis of these subgroups did not yield relevant differences in clinical characteristics or significant differences in survival rates (Table 3). However, patients with the anti-EGFR rechallenge showed a lower median OS in trend compared to patients with reintroduction, but the difference was not statistically significant (mOS 52.4 vs. 66.0, n.s.) (Figure 4). Similarly, median PFS for the last anti-EGFR-based therapy showed a trend towards a shorter mPFS in the rechallenge patients compared to the reintroduction patients without statistical significance (3.68 vs. 7.33 months, n.s.).

## 4. Discussion

In the present study, we aimed to assess the clinical implementation and utility of anti-EGFR retreatment strategies in real-world CRC patients. For patients with disease progression in second-, third-, or fourth-line treatment, therapy options are limited; hence, re-exposure to previously utilized agents is a common strategy in oncological practice [22]. For example, oxaliplatin is frequently reintroduced after prior stop-and-go strategies or discontinuation due to limited peripheral sensory neuropathy. Since anti-EGFR-based first- and second-line treatments have been commonly established in clinical practice, retreatment strategies, namely rechallenge and reintroduction, are increasingly employed in real-world oncology, although available evidence is limited and robust data from larger prospective trials regarding the efficacy of these retreatment strategies are lacking. Initially, a single-arm phase II prospective trial enrolled 39 patients to evaluate response rates after re-exposure to a cetuximab-based rescue therapy and prior progression on anti-EGFR treatment [19]. This study concluded that re-exposure might be a usable approach to broaden limited treatment options, but did not yield decisive results. Subsequently, a randomized phase II study (CAPRI-GOIM) assessed chemotherapy with FOLFOX plus cetuximab re-exposure compared to FOLFOX only [23]. The study observed a significantly longer PFS in cetuximab-retreated patients but no statistically significant difference in overall survival [23]. The prospective single-arm CRICKET study, a proof-of-concept trial, assessed whether rechallenge of cetuximab plus irinotecan was efficacious as a third-line treatment for patients with a *RAS* and *BRAF* wild-type, showing that anti-EGFR re-exposure indeed had activity in mCRC with acquired anti-EGFR resistance [18]. More recently, the single-arm phase II CAVE trial (cetuximab rechallenge plus avelumab) enrolled 77 mCRC patients, concluding that cetuximab plus avelumab are effective treatment strategies with manageable toxicity profiles [24]. The prospective CHRONOS trial recently demonstrated that liquid biopsy-driven rechallenge strategies can be feasible to improve clinical management [25]. However, while these prospective studies have been providing important evidence in regard to treatment efficacy and tolerability, due to their single-arm prospective design they also have major limitations, mostly generating preliminary evidence which is pending validation in larger phase III trials.

In this retrospective study, we presented additional real-world data on retreatment strategies derived from clinical practice. First, our data demonstrated anti-EGFR re-exposure to be associated with a distinctly higher OS, although only with borderline significance (*p* = 0.06), and high response rates to retreatment. Nevertheless, considering the specific clinical preconditions and subsequently limited number of patients in monocentric real-world subpopulations, the authors believe that a higher number of enrolled patients would further bolster the robustness of these findings. While mortality rates in real-world studies can inherently be affected by the retrospective nature of their design, our findings are largely in line with previous publications, which also indicated clinical benefits for patients re-exposed to anti-EGFR [17,26]. However, despite these previously published studies, available data to date do not reliably distinguish between outcomes between the anti-EGFR rechallenge and reintroduction settings. On the one hand, prospective trials did (I) only include patients with progression under EGFR therapy or (II) did not stratify study outcomes for rechallenge or reintroduction, respectively [18,19,23]. On the other hand, a limited number of retrospective studies has provided data on these retreatment strategies, but, notably, displayed some heterogeneity in definitions. While in some retrospective analyses, reintroduction was defined by a timed cut-off from the last anti-EGFR administrations (i.e., >3 months) [27], other studies defined reintroduction by the therapy sequence [20]. In the present study, we adapted a clear distinction based on the therapy sequence, according to which all patients assigned to the re-exposure group (either rechallenge or reintroduction) had received one or more intervening non-EGFR therapy block(s) prior to retreatment. Of note, the most frequent chemotherapy observed in our study cohort at re-exposure was FOLFIRI, while the most used anti-EGFR agent was cetuximab. To assess whether the reason for prior anti-EGFR treatment discontinuation affects clinical outcomes in our real-world cohort, we stratified patients for either rechallenge or reintroduction, as described earlier [17].

Noteworthy, previous retrospective studies have reported heterogeneous outcomes when assessing anti-EGFR retreatment strategies. While some found similar outcomes in patients receiving rechallenge and reintroduction [27], Karani et al. observed a significantly higher PFS in patients receiving reintroduction in their respective study cohort of 68 patients [26]. This study indicated that patients without the acquired anti-EGFR resistance, and hence no progression under first- or second-line therapy (reintroduction), might have a better anti-EGFR response to re-exposure. Although we cannot corroborate these findings definitively, our data show a distinct trend of higher OS and PFS in patients receiving reintroduction compared to rechallenge. The assumption that the acquired anti-EGFR resistance upon first anti-EGFR exposure could constitute a predictor for worse anti-EGFR response to re-exposure appears to be self-evidently in line with general paradigms, but clinical evidence is scarce and the findings discussed above need to be confirmed in prospective trials.

Of note, multivariate analysis in our real-world mCRC cohort confirmed that the primary CRC resection status was significantly associated with higher OS. Interestingly, in patients with anti-EGFR therapy, those receiving primary tumor resection and anti-EGFR re-exposure showed the highest OS among all groups [28]. However, survival rates among unresectable patients are commonly affected by selection bias, since those patients often present in highly advanced stages beyond operability at initial diagnosis. Therefore, survival rates derived from real-world data are frequently confounded by severity of disease.

Due to the retrospective nature, this study has several limitations. As our data demonstrated, indication for anti-EGFR re-exposure in clinical practice does not appear too frequently and, moreover, choices in treatment regimens upon patients’ disease progression are highly specific and consider a variety of patient- and context-sensitive factors. Therefore, the included patients into real-world databases are inherently heterogeneous in the clinical setting and management. Due to the time period of data collection, some patients were included in this study cohort before the results of the Fire-3 phase III trial (AIO KRK-0306) were published, which ultimately rendered RAS mutation analysis mandatory [29]. For this reason, we cannot provide comprehensive data on mutation analysis as well as HER2 status, particularly in regard to early recruited patients. In regard to more general limitations, the range of possible chemotherapeutic and anti-EGFR agents available results in a heterogeneous landscape of treatment combinations. As many with real-world studies assessing specific clinical conditions, we admittedly found our rechallenge and reintroduction sub-cohorts to be rather small. However, the clinical data presented in this study are largely in line with previously published clinical features of respective mCRC cohorts, including clinical characteristics and survival rates, even in the rather small anti-EGFR retreatment subgroups.

## 5. Conclusions

In summary, the present study provides clinical evidence underscoring that real-world mCRC patients most likely benefit from anti-EGFR re-exposure independently from the reason for prior discontinuation. Although rechallenge and reintroduction have emerged as valuable treatment options for mCRC patients in recent years, these findings should be confirmed by larger prospective randomized trials in the future. However, as patients treated with anti-EGFR re-exposure show a favorable overall prognosis, we consider the administration of more than one line of EGFR directed therapy a promising tool to improve patients’ survival.

## Figures and Tables

**Table 1:** General patient characteristics in the overall mCRC cohort.

Parameters	No Anti-EGFR Therapy (*n* = 381)	Anti-EGFR Therapy (*n* = 143)	*p*-Value
Patient characteristics			
Age at first diagnosis (mean, SD)	63.57 (12.28)	58.83 (12.69)	<0.001
Female (patients, %)	167 (43.8)	51 (35.7)	0.19
Study trial inclusion (patients, %)	69 (18.1)	59 (41.3)	<0.001
Survival			
Overall survival (median, 95% CI)	39.6 (33.0–46.0)	38.3 (32.8–48.4)	0.8
Progression-free survival (median, 95% CI)	12.5 (10.61–15.2)	10.1 (8.61–12.9)	0.2
Primary tumor localization			
Left-sided CRC (patients, %)	241 (63.4)	114 (79.7)	<0.001
Operation			
Primary CRC resection (patients, %)	183 (48.0)	69 (48.3)	1.0
Metastectomie (patients, %)	11 (2.9)	7 (4.9)	
Stage at first diagnosis (UICC, patients, %)			0.15
I	2 (0.5)	0 (0)	
II	19 (5.0)	2 (1.4)	
III	50 (13.1)	13 (9.1)	
IV	310 (81.4)	125 (87.4)	
n.a.	0 (0)	3 (2.1)	
Metastasis at first diagnosis			
Synchron metastasis (patients, %)	310 (81.4)	125 (87.4)	0.22
1 synchronous metastasis (patients, %)	236 (61.9)	99 (69.2)	
≥2 synchronous metastasis (patients, %)	74 (19.4)	26 (18.2)	
Site of metastasis			0.028
Liver (patients, %)	194 (50.9)	95 (66.4)	
Lung (patients, %)	48 (12.6)	16 (11.2)	
Other (patients, %)	92 (24.1)	23 (16.1)	
n.a.	47 (12.3)	9 (6.3)	
Tumor marker			
CEA (mean, SD)	567 (2057)	792 (2011.1)	0.52

**Table 2:** Characteristics of patients receiving anti-EGFR treatment.

Parameters	Anti-EGFR-Based Treatment, No Re-Exposure (*n* = 110)	Anti-EGFR Re-Exposure (*n* = 33)	*p*-Value
Patient characteristics			
Age at first diagnosis (mean, SD)	59.6 (12.9)	56.1 (11.7)	<0.001
Female (patients, %)	37 (33.6)	14 (42.42)	0.41
Study trial inclusion (patients, %)	40 (36.4)	19 (57.6)	<0.05
Survival			
Overall survival (median, 95% CI)	35.4 (26.6–42.6)	56.0 (37.5–77.1)	0.06
Stage at first diagnosis (UICC, patients, %)			0.53
0	0 (0)	0 (0)	
I	0 (0)	0 (0)	
II	0 (0)	2 (6.1)	
III	10 (9.1)	3 (9.1)	
IV	94 (85.5)	28 (84.8)	
unknown	6 (5.5)	0 (0)	
Primary tumor localization			
Left-sided CRC (patients, %)	87 (79.1)	27 (81.9)	0.81
Operation			
Primary CRC resection (patients, %)	83 (75.5)	22 (66.7)	0.17
R0 resection (patients, %)	72 (91.1)	19 (95.0)	0.85
Number of pos. LK (mean, SD)	28.98 (27.54)	23.17 (17.17)	0.38
Palliative treatment intention (patients, %)	40 (48.8)	12 (54.5)	0.81
Metastectomie (patients, %)	4 (3.6)	3 (9.1)	0.44
Metastasis at first-line			0.5
Metastasis, 1 organ	63 (60.6)	21 (63.6)	
Metastasis, ≥2 organs, not peritoneal	27 (24.6)	6 (18.2)	
Metastasis, periteoneal	2 (1.9)	0 (0)	
Site of metastasis at first-line			0.46
Liver (number, %)	73 (66.4)	22 (66.7)	
Lung (number, %)	10 (9.1)	6 (18.2)	
Other (number, %)	20 (18.2)	3 (9.1)	
Tumor marker			
CEA (mean, SD)	686.41 (1969.4)	1094.22 (2187.91)	0.55
CA 19–9 (mean, SD)	2999.0 (4507.8)	453.80 (739.64)	0.24

**Table 3:** Characteristics of patients with anti-EGFR rechallenge and anti-EGFR reintroduction.

Parameters	Rechallenge (*n* = 21)	Reintroduction (*n* = 12)	*p*-Value
Patient characteristics			
Age at first diagnosis (mean, SD)	56.1 (13.9)	56.1 (10.6)	0.88
Female (patients, %)	7 (33)	7 (58.3)	0.31
Study trial inclusion (patients, %)	14 (66.7)	5 (41.7)	0.27
Survival			
Overall survival (median, 95% CI)	52.4 (28.6–84.3)	66.0 (35.7–N.A.)	0.47
Stage at first diagnosis (UICC, patients, %)			0.73
0	0 (0)	0 (0)	
I	0 (0)	0 (0)	
II	1 (4.8)	1 (8.3)	
III	2 (9.5)	1 (8.3)	
IV	18 (85.7)	10 (83.3)	
Primary tumor localization			
Left-sided CRC (patients, %)	18 (85.7)	9 (75)	0.44
Primary tumor resection			
yes	12	10	0.38
R0 resection	11 (91.7)	8 (88.9)	0.96
Number of pos. LK (mean, SD)	25.95 (22.6)	21.1 (12.7)	0.63
Palliative treatment intention (patients, %)	6 (60)	6 (50)	
Metastasis at first-line			0.7
Metastasis, 1 organ	13 (61.9)	8 (66.7)	
Metastasis, ≥2 organs, not peritoneal	4 (19.0)	2 (16.7)	
Metastasis, periteoneal	0	0	
Site of metastasis at first-line			0.78
Liver	8 (38.1)	5 (41.7)	
Lung	2 (9.5)	0 (0)	
Lymph nodes	1 (4.8)	0 (0)	
other	1 (4.8)	0 (0)	
Tumor marker			
CEA (mean, SD)	1361.3 (2500.3)	293.1 (199.54)	0.617
CA19-9 (mean, SD)	551.3 (816.2)	64 (n/a)	n.a.

## Data Availability

The data presented in this study are available on request from the corresponding author.

## Supplementary Materials

The following supporting information can be downloaded at: https://www.mdpi.com/article/10.3390/cancers14071641/s1, Figure S1: Kaplan-Meier estimator for overall (left) and progression-free (right) survival in the study cohort of all CRC patients (*n* = 524). Table S1. Multivariate regression in the cohort of all mCRC patients (*n* = 524), Table S2. Response rates to first anti-EGFR exposure and anti-EGFR retreatment, Table S3. Multivariate regression in the cohort of anti-EGFR-treated patients (*n* = 143), Table S4. Treatment sequence of patients receiving anti-EGFR rechallenge and anti-EGFR reintroduction, STROBE Statement—Checklist of items that should be included in reports of cohort studies.

## Abbreviations

Anti-EGFR	anti-epidermal growth factor receptor monoclonal antibody
BRAF	B rapidly accelerated fibrosarcoma gene
CA19-9	carbohydrate antigen 19-9
CEA	carcinoembryonic antigen
CR	complete response
CT	chemotherapy
EGFR	epidermal growth factor receptor
FOLFIRI	chemotherapy regime containing folinic acid (FOL), fluorouracil (F), and irinotecan (IRI)
FOLFOX	Chemotherapy regime containing folinic acid (FOL), fluorouracil (F), and oxaliplatin (OX)
FU	follow-up
KRAS	Kirsten rat sarcoma virus gene
LK	lymph node
mCRC	metastatic colorectal carcinoma
NRAS	Neuroblastoma RAS viral oncogene homolog
OS	overall survival
PD	progressive disease
PFS	progression-free survival
PR	partial response
R0 resection	resection margin (0) without cancer cells microscopically at the primary tumour site
RAS	Rat sarcoma proto-oncogene
RMS	remission-free survival
SD	stable disease
UICC	Union for International Cancer Control
